# Immobilization and Characterization of a New Regioselective and Enantioselective Lipase Obtained from a Metagenomic Library

**DOI:** 10.1371/journal.pone.0114945

**Published:** 2015-02-23

**Authors:** Robson Carlos Alnoch, Viviane Paula Martini, Arnaldo Glogauer, Allen Carolina dos Santos Costa, Leandro Piovan, Marcelo Muller-Santos, Emanuel Maltempi de Souza, Fábio de Oliveira Pedrosa, David Alexander Mitchell, Nadia Krieger

**Affiliations:** 1 Departamento de Bioquímica e Biologia Molecular, Universidade Federal do Paraná, Cx. P. 19046 Centro Politécnico, Curitiba 81531–980, Paraná, Brazil; 2 Instituto Federal do Paraná–Campus Irati, Irati 84500–000, Paraná, Brazil; 3 Agência Tecpar de Inovação, Instituto de Tecnologia do Paraná—Tecpar, Curitiba 81350–010, Paraná, Brazil; 4 Departamento de Química, Universidade Federal do Paraná, Cx. P. 19081 Centro Politécnico, Curitiba 81531–980, Paraná, Brazil; Wageningen University, NETHERLANDS

## Abstract

In previous work, a new lipase and its cognate foldase were identified and isolated from a metagenomic library constructed from soil samples contaminated with fat. This new lipase, called LipG9, is a true lipase that shows specific activities that are comparable to those of well-known industrially-used lipases with high activity against long-chain triglycerides. In the present work, LipG9 was co-expressed and co-immobilized with its foldase, on an inert hydrophobic support (Accurel MP1000). We studied the performance of this immobilized LipG9 (Im-LipG9) in organic media, in order to evaluate its potential for use in biocatalysis. Im-LipG9 showed good stability, maintaining a residual activity of more than 70% at 50°C after incubation in n-heptane (log *P* 4.0) for 8 h. It was also stable in polar organic solvents such as ethanol (log *P* -0.23) and acetone (log *P* -0.31), maintaining more than 80% of its original activity after 8 h incubation at 30°C. The synthesis of ethyl esters was tested with fatty acids of different chain lengths in *n*-heptane at 30 °C. The best conversions (90% in 3 h) were obtained for medium and long chain saturated fatty acids (C8, C14 and C16), with the maximum specific activity, 29 U per gram of immobilized preparation, being obtained with palmitic acid (C16). Im-LipG9 was *sn*-1,3-specific. In the transesterification of the alcohol (*R,S*)-1-phenylethanol with vinyl acetate and the hydrolysis of the analogous ester, (*R,S*)-1-phenylethyl acetate, Im-LipG9 showed excellent enantioselectivity for the *R*-isomer of both substrates (*E*> 200), giving an enantiomeric excess (*ee*) of higher than 95% for the products at 49% conversion. The results obtained in this work provide the basis for the development of applications of LipG9 in biocatalysis.

## Introduction

Lipases (glycerol ester hydrolases EC 3.1.1.3) are carboxylesterases that normally catalyze the hydrolysis of long and short-chain acyl esters in aqueous media [1]. However, when used in water-restricted media, they can catalyze esterification and transesterification reactions, with high regio- and enantioselectivity [1,2]. Due to these properties, lipases have been used in organic media for the synthesis of biodiesel [3,4] and of chiral compounds that have applications in the chemical, pharmaceutical and cosmetic industries [5,6].

A major barrier to the industrialization of lipase-catalyzed processes is their low productivity when compared to chemical routes of synthesis. Additional disadvantages are the high costs of the enzymes and their low stability in water-restricted media. In fact, denaturation of the lipase is frequently a serious problem in the case of biodiesel synthesis, where one of the substrates is a short-chain alcohol, such as methanol or ethanol [7,8]. It is therefore necessary to obtain new lipases that have a high and stable activity in organic solvents, including short-chain alcohols.

Over the years, many researchers have tried to obtain new lipases either by modifying current lipases using techniques of molecular biology or by screening samples obtained from natural or polluted environments [9–11]. One promising approach is the screening for new enzymes in metagenomic libraries [12]. These libraries contain genetic material that has been extracted from environmental samples and cloned into hosts such as *Escherichia coli*. This technique is particularly promising for the isolation of new lipases: since it does not involve isolation and cultivation of whole microorganisms, it enables lipases to be obtained from so-called “non-culturable” microorganisms [12,13].

Over the last decade, several lipases and esterases have been obtained from metagenomic libraries derived from soil or sediments collected from mangroves [14], Atlantic Forest [15], a compost heap [16,17], an oil-contaminated area [18] and a hot spring [19]. However, relatively little work has been done to characterize the potential of these enzymes for catalyzing reactions in organic media [20] and the work that has been undertaken has involved the use of free enzymes. This is in spite of the fact that, in commercial processes, lipases are typically used in immobilized form, since this makes it easy to recover the enzyme from the reaction medium and reuse it.

In previous work, we identified a new lipase, LipG9, in a metagenomic library constructed from soil samples contaminated with animal fat [18]. LipG9 was overexpressed and preliminary studies were performed with the purified enzyme [21]. It was shown that LipG9 was active only when co-expressed with its foldase (LifG9) and that after the purification process, LipG9 and LifG9 were co-eluted and remained complexed. The specific activities of LipG9 in the hydrolysis of long-chain triglycerides were comparable to those of several well-known commercial lipases, such as the lipases from *Thermomyces lanuginosus*, *Rhizopus oryzae* and *Rhizomucor miehei* [21]. These features suggested that LipG9 may have potential for use in biocatalysis. However, more studies are needed to confirm this potential.

In the present work, we immobilize LipG9, co-expressed with its foldase, on the polypropylene support Accurel MP1000 and study its behavior in water-restricted media. This immobilized complex (hereafter referred to as Im-LipG9) has remarkable stability in polar and non-polar organic solvents, catalyzes ester synthesis with various saturated and unsaturated fatty acids and is regioselective (being *sn*-1,3-specific) and enantioselective. These properties of LipG9 justify future efforts towards the development of its applications in biocatalysis.

## Materials and Methods

### Reagents and Materials

*E*. *coli* BL21 (DE3), pET28a (+) (Novagen, WI, USA), pT7–7 (USB, OH, USA) and IPTG (Isopropyl β-D thiogalactopyranoside) (Invitrogen Life Technologies, CA, USA) were used in the recombinant protein expression system. The HiTrap Chelating Sepharose HP column (HiTrap) was purchased from GE Healthcare (Uppsala, Sweden). Activity assays involved the substrates triolein (C18, purities of 65% and 99%), tricaprylin (C8, 99%), caprylic acid (C8:0, 99%), lauric acid (C12:0, 99%), myristic acid (C14:0, 99%), palmitic acid (C16:0, 99%), stearic acid (C18:0, 99%), oleic acid (C18:1, 90%) and linoleic acid (C18:2, 99%) (Sigma-Aldrich, St. Louis, MO, USA). For the immobilization, polypropylene beads (Accurel MP 1000, Membrane GmbH, BY, Germany) were used, with the following characteristics: surface area of 55.985 m^2^ g^-1^, particle density of 1.993 g cm^-3^ and particle diameter <1500 mm. All other reagents and substrates used were of analytical grade.

### Overexpression and purification of LipG9

Both the overexpression and the purification of the complex Lip-LifG9 were performed according to Martini et al. [21]. *E*. *coli* BL21(DE3) cells carrying the plasmids pET28a-*lipG9* and pT7–7-*lifG9* were grown in 200 mL of LB medium at 37°C until an OD_600_ of 0.5 and induced by the addition of IPTG to a final concentration of 0.5 mM. The induced culture was incubated for a further 16 h at 20°C before harvesting of the cells by centrifugation (3000×*g* for 5 min) at 4°C. The cell pellet was resuspended in 35 mL of lysis buffer (50 mM Tris-HCl pH 8.0, 500 mM NaCl, 10 mM imidazole, 10 mM β-mercaptoethanol, 10% (v/v) glycerol, 0.25% (w/v) Nonidet P-40) and disrupted by ultrasonication in an ice bath (10 cycles of 60-s pulses, 90 W, with 30-s intervals), using a SONICATOR XL 2020 (Heat systems-Ultrasonics Inc., NY, USA). The crude extract was then centrifuged at 30000×*g* for 30 min at 4°C to pellet the cell debris. The supernatant containing the His-tagged LipG9 complexed with its foldase LifG9 was loaded onto a HiTrap column, previously loaded with Ni^2+^ and equilibrated with lysis buffer, using an ÄKTA basic (Uppsala, Sweden) chromatography system. The column was washed with 5 volumes of the lysis buffer and then with 5 volumes of elution buffer (50 mM Tris-HCl pH 8.0, 500 mM NaCl, 10 mM imidazole, 10% (v/v) glycerol). The complex Lip-LifG9 was eluted with an increasing gradient of imidazole (up to 1 M) in elution buffer. The elution of the complex was monitored at 280 nm and the protein fractions were analyzed by SDS-PAGE, pooled, dialyzed (50 mM Tris-HCl pH 8.0, 150 mM NaCl, 10 mM CaCl_2_, 20% (v/v) glycerol) and stored at 4°C until use. Hereafter, Lip-LifG9 is referred to simply as LipG9.

### Gel electrophoresis and protein determination

Electrophoresis of protein samples (SDS-PAGE) was done with 12% (w/v) gel [22]. The gel was stained with Coomassie Brilliant Blue R-250 and destained with methanol/acetic-acid/water (5/1/4 v/v/v). Protein content was determined by the method of Smith [23], using the Pierce BCA Protein Assay Kit (Pierce Biotechnology, IL, USA) with bovine serum albumin as the standard.

### Lipase activity assay

The hydrolysis activity in aqueous solution was determined by titration using an automatic titrator pHStat (Metrohm 718 Stat Titrino) [24]. The substrate emulsions consisted of 67 mM triacylglycerol, 3% (w/v) gum arabic, 2 mM CaCl_2_, 2.5 mM Tris-HCl pH 8.0 and 150 mM NaCl, dispersed in distilled water [25]. The enzyme was added to 20 mL of the emulsion under magnetic stirring (300 rpm) at 30°C and the reaction was followed for 5 min. One unit of hydrolytic activity in aqueous medium corresponds to the release of 1 μmol of fatty acid per minute, under the assay conditions.

The hydrolytic activity in organic medium was determined by adding 5 mL of a reaction medium containing 4.9 mL of *n*-heptane, 70 mM triolein and 2% (v/v) distilled water to a 25-mL Erlenmeyer flask. The reaction was started by the addition of either free (Fr-LipG9) or immobilized LipG9 (Im-LipG9). The mixture was then incubated in an orbital shaker at 200 rpm and 40°C. At fixed intervals, 100-μL samples of the mixture were collected and analyzed for residual free fatty acids by the Lowry-Tinsley method [26]. One unit of hydrolytic activity in organic medium corresponds to the release of 1 μmol of fatty acid per minute, under the assay conditions.

### Immobilization of LipG9

LipG9 was immobilized by physical adsorption on Accurel MP 1000 according to Al-Duri and Yong [27]. The support was wetted with ethanol solution (50% (v/v) in water) for 30 min, then washed with distilled water and filtered. To study the effect of the protein loading (i.e. mg of protein offered for immobilization per g of support), 0.1 g of the support and 10 mL of enzyme solution with the stated protein concentration were added to a 25-mL Erlenmeyer flask and incubated in an orbital shaker at 150 rpm and 4°C. The time course of immobilization was evaluated by determining the residual activity and protein concentration in aliquots of the supernatant removed over time (0–48 h). Finally, the immobilized enzyme was removed from the mixture by filtration with qualitative filter paper (Whatman n° 1), dried in a vacuum desiccator for 16 h and stored at 4°C.

The immobilization efficiency (IE, %) was calculated as:

$$\mathrm{IE}=\frac{A_{i}-A_{f}}{A_{i}}\times 100\%$$ (1)

where *A*_*i*_ is the hydrolytic activity (U) of the enzyme solution before immobilization and *A*_*f*_ is the hydrolytic activity (U) remaining in the supernatant at the end of the immobilization procedure [28].

The retention of activity (R, %) was calculated as:

$$R=\frac{A_{O}}{A_{T}}\times 100\%$$ (2)

where *A*_*O*_ is the observed hydrolytic activity in organic medium of the immobilized preparation (U g^-1^ of support) and *A*_*T*_ is the theoretical activity of the immobilized preparation (U g^-1^ of support), calculated based on the amount of activity removed from the supernatant during the immobilization procedure [28].

In order to confirm the immobilization of LipG9 in Accurel MP 1000, proteins were desorbed from the support by incubating Im-LipG9 in a solution of SDS 10% (w/v) at 180 rpm for 2 h, at 40°C.

### Thermal stability of LipG9

The thermal stabilities of Fr-LipG9 and Im-LipG9 were assessed by incubation in a water bath for 8 h at temperatures from 30 to 60°C. Fr-LipG9 (0.045 mg) was incubated in 300 μL buffer (50 mM Tris-HCl pH 8.0, 150 mM NaCl, 2 mM CaCl_2_) and its residual activity was evaluated in aqueous medium by the titrimetric method (pHStat). Im-LipG9 (20 mg) was incubated in 1 mL of *n*-heptane (to prevent desorption of the enzyme from the support) and its residual activity was evaluated by hydrolysis of triolein in organic medium. The residual activities were calculated in relation to controls that were treated identically, but without incubation.

### Stability of immobilized LipG9 in organic solvents

The effect of organic solvents on the stability of Im-LipG9 was determined using the following solvents (with their log *P* values): *n*-heptane (4), hexane (3.5), cyclohexane (3.2), toluene (2.5), *tert*-butanol (1.45), *n*-butanol (0.8), ethyl acetate (0.68), tetrahydrofuran (0.46), *n*-propanol (0.25), ethanol (-0.23), acetone (-0.31), acetonitrile (-0.33), methanol (-0.76) and dimethylsulfoxide (-1.3). Im-LipG9 (20 mg) was incubated in each solvent for 8 h at 30°C, without agitation. The solvent was then removed by filtration through filter paper (Whatman n° 1) and Im-LipG9 was then dried in a vacuum desiccator (16 h, at 4°C) and its residual activity was evaluated by the hydrolysis of triolein in organic medium. The residual activities were calculated relative to a control performed without pre-incubation in the solvents.

### Application of immobilized LipG9 in ester synthesis

The performance of Im-LipG9 in the synthesis of ethyl-oleate in organic medium was compared to that of Fr-LipG9. Given that lyophilized Fr-LipG9 did not have activity when added directly to the organic medium, a purified aqueous extract containing the enzyme was added instead. The reaction medium (5 mL) contained 4.9 mL of *n*-heptane, 70 mM oleic acid and 210 mM ethanol, in a 25-mL Erlenmeyer flask. The reaction was started by the addition of either Fr-LipG9 or Im-LipG9, followed by incubation in an orbital shaker at 200 rpm and 40°C. At fixed intervals, 100-μL samples of the mixture were collected and analyzed for residual free fatty acids by the Lowry-Tinsley method [26]. The reaction yield and the activity of esterification of Im-LipG9 were calculated based on the consumption of free fatty acids. One unit of lipase esterification activity (U) corresponds to the consumption of 1 μmol of fatty acid per minute, under the assay conditions.

The effects of the chain length and the degree of unsaturation of the fatty acid were evaluated. The esterification reactions were performed as described above, except that various fatty acids were used: caprylic (C8:0), lauric (C12:0), myristic (C14:0), palmitic (C16:0), stearic (C18:0) and linoleic (C18:2).

### Evaluation of the regioselectivity of LipG9

In order to determine the regioselectivity of Fr-LipG9 and Im-LipG9, an assay was carried out, in organic medium, using triolein (99% purity, Sigma-Aldrich, MO, USA) as the substrate, and the reaction products were examined by thin-layer chromatography (TLC), according to Sugihara et al. [29]. The reaction medium was composed of 20 mM triolein, 0.1 mL distilled water (2%, v/v) and 4.9 mL of *n*-heptane. The reaction was started by addition of 30 mg of Im-LipG9 or 50 μL (containing 0.07 mg protein) of the solution of Fr-LipG9. The mixture was kept in an orbital shaker for 30 min at 30°C and 180 rpm. Aliquots (2 μL) were applied on a silica gel 60 plate (Merck, Darmstadt, HE, Germany) and developed with a 95:4:1 (v/v) mixture of chloroform, acetone and acetic acid. The spots were visualized using a saturated iodine chamber and their R_f_ values were compared with those of the standards 1 (2)-monoolein, oleic acid, 1,2 (2,3)-diolein, 1,3-diolein and triolein (Sigma-Aldrich, MO, USA).

### Evaluation of the enantioselectivity of Im-LipG9

**Fig 1:**
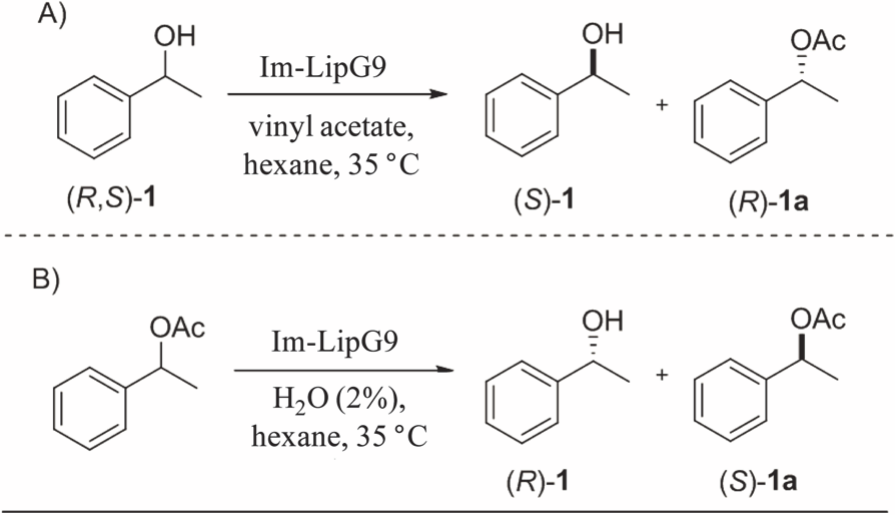
Model reactions for the evaluation of the enantioselectivity of immobilized LipG9 (Im-LipG9) in (A) transesterification and (B) hydrolysis reactions.

The enantioselectivity of Im-LipG9 was evaluated in two reactions: (1) the transesterification of (*R*,*S*)-1-phenyl-1-ethanol [(*R*,*S*)-**1**] with vinyl acetate as the acyl moiety donor (Fig. 1A); and (2) the hydrolysis of (*R*, *S*) 1-phenylethyl acetate [(*R*,*S*)-**1a**], with H_2_O as the nucleophile (Fig. 1B). Both reactions were carried out with *n*-hexane as the solvent. The secondary alcohol [(*R*,*S*)-**1**] and its respective ester [(*R*,*S*)-**1a**], both racemic, were chosen because they have a significant difference between the sizes of the groups at the stereocenter (i.e. phenyl and methyl groups). According to Kazlauskas’s rule, substrates with substituents of significantly different sizes are better resolved by lipases [30]. Compounds (*R*,*S*)-**1** and (*R*,*S*)-**1a** were synthesized according to methodologies that are already described in the literature and characterized by spectroscopic techniques (see S1 Text).

For the transesterification reaction, 100 mg of Im-LipG9 (corresponding to 22 U of triolein-hydrolyzing activity in organic medium) was added to a solution of (*R*,*S*)-1-phenyl-1-ethanol (12 mg, 0.1 mmol) and vinyl acetate (35 mg, 4 mmol) in *n*-hexane (2 mL). The reaction was carried out at 35°C and 150 rpm. Samples were removed and analyzed by gas chromatography in a GC-2010 chromatographer (Shimadzu Co., Kyoto, Japan), equipped with a hydrogen flame ionization detector, and a chiral column CP Chirasil-DEX CB (25 m × 0.25 mm diameter, 0.25 μm film thickness). A sample of 1 μL was injected with a split ratio of 1:50, using N_2_ as the carrier gas. The injector and detector were set at 220°C. The oven program was as follows: initial value of 110°C, with heating at 1°C min^-1^ to 120°C. Retention times were: (*R*)-**1** 3.63 min, (*S*)-**1** 3.93 min, (*S*)-**1a** 4.43 min and (*R*)-**1a** 4.68 min.

For the hydrolysis reaction, 100 mg of Im-LipG9 (corresponding to 22 U of triolein-hydrolyzing activity in organic medium) was added to a reaction medium containing (*R*,*S*) 1-phenylethyl acetate (16 mg, 0.1 mmol) and water (2.2 mmol, 4 mg, corresponding to 2%) in *n*-hexane (2 mL). The reaction was carried out at 35°C and 150 rpm. Samples were removed and analyzed by GC as described above for the transesterification reaction.

The absolute configurations of the enantiomers, of both the alcohol and the ester, were attributed indirectly by comparing the retention times of the substrates and products involved in the reactions catalyzed by Im-LipG9 with the retention times of the enantiomers produced in the same reactions catalyzed by the lipase B of *Candida antarctica* (CALB, Novozymes, Denmark). The (*R*)-enantiopreference of CALB for these compounds is already described in the literature [31,32].

The enantiomeric excesses of substrate (*ee*_*s*_, %) and product (*ee*_*p*_, %) were determined based on the differences in the relative percentages of each enantiomer:

$$ee=\left[\frac{R-S}{R+S}\right]\times 100\%$$ (3)

where *R* is the concentration of the appropriate R-enantiomer and *S* is the concentration of the appropriate S-enantiomer.

The conversion (*c*, %) and enantioselectivity (*E*) were calculated from the values of *ee*_*s*_ and *ee*_*p*_ according to Chen et al. [33]:

$$c=\frac{ee_{s}}{\left(ee_{s}+ee_{p}\right)}\times 100\%$$ (4)

$$E=\frac{\ln\left[\left(1-c\right)\left(1-ee_{s}\right)\right]}{\ln\left[\left(1-c\right)\left(1+ee_{s}\right)\right]}$$ (5)

## Results

### Purification and immobilization of LipG9

**Fig 2:**
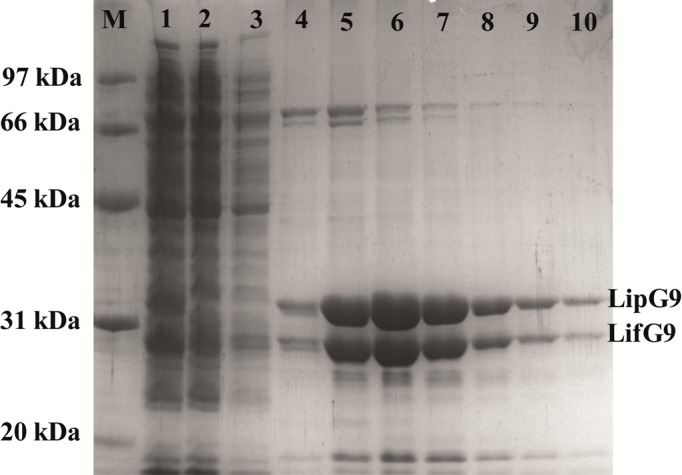
SDS-PAGE analyses of the fractions eluted from the nickel column. The lanes were loaded as follows: lane M, protein molecular weight standards; lane 1, supernatant of the bacterial cell lysate; lane 2, crude cell extract (precipitated); lane 3, column wash; lane 4–10, eluted fractions during purification on nickel column (Ni^2+^). Proteins were stained with Coomassie brilliant blue R-250.

Protein bands corresponding to the molecular masses of LipG9 (32 kDa) and its foldase LifG9 (24 kDa) were observed after SDS-PAGE of the eluate from the HiTrap column (see lanes 4 to 10 in Fig. 2). The eluted fractions were pooled and dialyzed in buffer containing 20% (v/v) glycerol with no imidazole. Table 1 summarizes the results of the purification step, showing an activity yield of 41% and a purification factor of 12. The specific hydrolytic activity in aqueous medium of the purified fraction of LipG9 (complexed with its foldase), used in further experiments, was 2634 U mg^-1^ with tricaprylin and 911 U mg^-1^ with triolein.

**Table 1:** Summary of the purification of the lipase LipG9 by affinity chromatography on nickel column.

Step	Volume (mL)	Total Protein ^a^ (mg)	Total Activity ^b^ (U)	Specific activity (Umg^-1^ protein)	Purification Factor	Activity Yield (%)
Crude extract	35	197.0	43550	221	1	100
Purified extract	9	6.7	17650	2634	12	41

^a^ Protein was determined by the method of Smith et al. [23].

^b^ A unit of activity (U) represents the production 1 μmol of fatty acid per min using tricaprylin as substrate in the titrimetric method using a pHStat, at 30°C for 5 min.

Protein loadings of 5, 10 and 15 mg g^-1^ were evaluated for the immobilization of LipG9 by physical adsorption on Accurel MP 1000. The best immobilization efficiency (IE 68%) and the highest specific hydrolytic activity in organic medium (66 U mg^-1^ of protein) were obtained for the loading of 5 mg g^-1^. For higher protein loadings (10 and 15 mg g^-1^), lower IE values were obtained (54% and 36%, respectively), whereas the specific activity of the immobilized preparation remained almost constant (43 and 46 U mg^-1^, respectively), indicating saturation of the support. For assays done with loadings varying from 1 to 4 mg g^-1^, Im-LipG9 showed low activity, in spite of the fact that all activity disappeared from the supernatant after the immobilization procedure (IE 100%, data not shown).

The activity retention values (R), calculated in comparison to the specific activity of the aqueous extract of Fr-LipG9 in organic medium (26 U mg^-1^), were above 100% for all immobilized preparations (Table 2). This shows that LipG9 was activated upon immobilization on Accurel MP 1000. The highest value of R, 249%, was obtained with the protein loading of 5 mg g^-1^. This loading was used for the characterization experiments.

**Table 2:** Principal parameters for immobilization of the lipase LipG9 on Accurel MP 1000.

Protein loading (mg g^-1^ support)	Efficiency of Immobilization ^a^ (%), IE	Lipase activity (U g^-1^ support)	Specific activity (U mg^-1^ protein)	Retention of activity ^b^ (%), R
5	68	219 ± 28 ^c^	66 ± 9	248
10	54	230 ± 6	43 ± 1	164
15	36	249 ± 22	46 ± 5	178

^a^ Calculated as the difference between the initial and final activities in the supernatant after immobilization using the titrimetric method (pHStat) and tricaprylin as substrate.

^b^ Retention of activity (%), measured as the ratio between the real activity (U g^-1^ support) of immobilized LipG9 in organic media, and theoretical activity of the immobilized LipG9 (U g^-1^ support), calculated from the difference in the activities measured in the supernatant at the beginning and end of the immobilization process.

^c^ SEM: Standard error of the mean.

**Fig 3:**
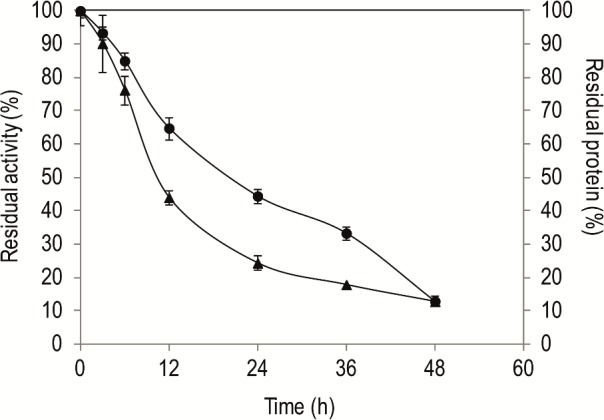
Kinetics of adsorption of LipG9 on Accurel MP1000. Key: (▲) Residual activity in the supernatant, determined with tricaprylin as the substrate, by the titrimetric method using a pHStat, at 30°C for 5 min; (●) Residual protein in the supernatant, determined by method of Smith et al. [23]. Adsorption was performed with an initial protein loading (mg protein per g support) of 5 mg g^-1^, at 4°C, 150 rpm.

The time course of LipG9 immobilization was followed in order to determine the best immobilization time (Fig. 3). There was no loss of activity in a control in which the enzyme solution was incubated for 48 h under the same conditions but without the support. The immobilization efficiency was higher (IE 88%) after 48 h as compared to 24 h (IE 68%). However, the specific activity and the retention of activity at 48 h were lower (52 U mg^-1^, R 152%) than at 24 h (66 U mg^-1^, R 249%) (Table 2). This result suggests that, from 24 h on, the surface of the support might already be saturated with protein and that at longer immobilization times the protein is absorbed in thicker layers that restrict activity of some of the enzyme due to mass transfer limitations. Based on this result, the time of 24 h was chosen for the immobilization of LipG9.

**Fig 4:**
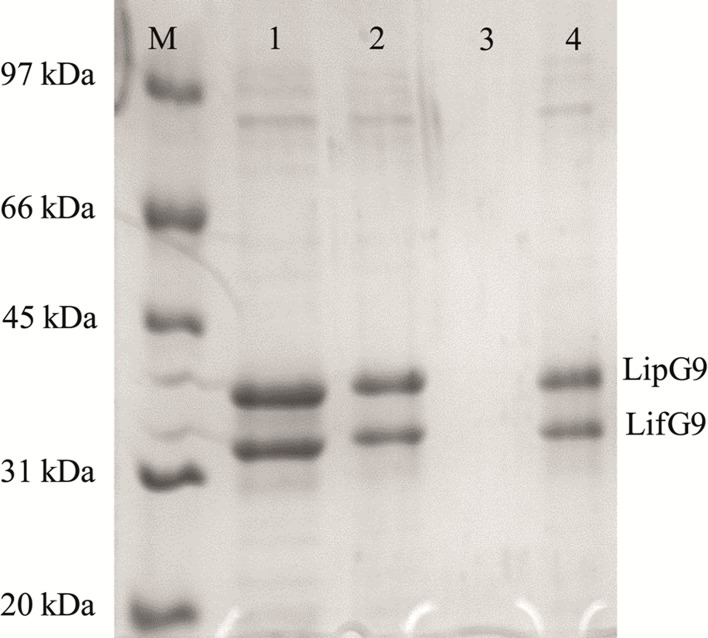
SDS-PAGE analyses of the fractions desorbed from the support Accurel MP 1000 after immobilization of LipG9. The lanes were loaded as follows: lane M, protein molecular weight standards; lane 1, purified LipG9; lane 2, supernatant at time 0 h, lane 3, supernatant after 48 h of immobilization; lane 4, fractions desorbed from Accurel MP 1000 after being washed with 10% (w/v) SDS solution. Proteins were stained with Coomassie brilliant blue R-250.

After 2 h incubation of the immobilized preparation in an SDS solution, SDS-PAGE of the supernatant gave bands at 32 kDa and 23 kDa (see lane 4 of Fig. 4). These bands correspond to LipG9 and LifG9, respectively. This result shows that the lipase was immobilized on the support with its foldase.

### Thermal stability of LipG9

**Fig 5:**
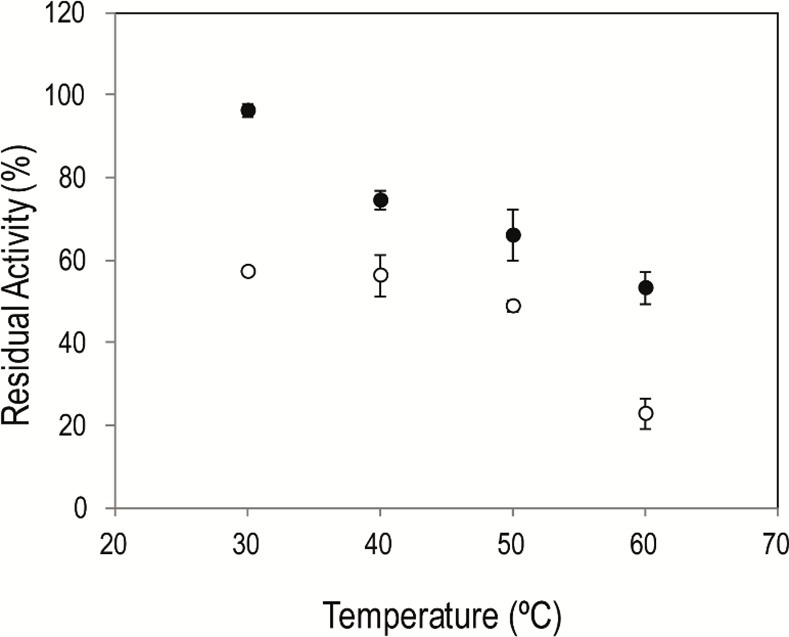
Thermal stability of free and immobilized LipG9. Key: (○) Residual activity of free enzyme (Fr-LipG9); (●) Residual activity of immobilized enzyme (Im-LipG9). Fr-LipG9 was incubated in 1 mL of buffer for 8 h. The residual activity of Fr-LipG9 was determined by the titrimetric method using a pHStat. Im-LipG9 was incubated in 1 mL of *n*-heptane for 8 h and its residual activity was measured by the hydrolysis of triolein in organic media. The error bars represent the standard error of the mean.

When incubated for 8 h, Im-LipG9 retained 100% of its activity at 30°C, more than 70% at 40°C and 50°C and around 50% at 60°C (Fig. 5). Under the same incubation conditions, the residual activity of Fr-LipG9 was approximately 50% at 50°C, but only 20% at 60°C. The thermal stability of LipG9 is therefore enhanced upon immobilization, as has been shown for other lipases [34–37]. This improved stability is due to interactions between the enzyme and the support [34], which confer a more rigid structure on the enzyme [38,39]. The higher thermal stability of Im-LipG9 could also be due, in part, to the fact that it was incubated in a hydrophobic organic solvent in the absence of free water: this can also make the enzyme more rigid and therefore less prone to denaturation [40].

### Stability of Im-LipG9 in organic solvents

We investigated the stability of Im-LipG9 in polar and non-polar solvents, with log *P* values ranging from -1.30 to 4.0. Im-LipG9 retained over 80% of its activity after 8 h incubation in *n*-heptane, *n*-hexane, toluene, tetrahydrofuran, acetone and ethanol (Table 3). Low residual activity (30%) was observed in methanol and DMSO completely denatured the enzyme. Normally, lipases are more active and stable in non-polar solvents with log *P*>2.5 [41,42]. Polar solvents, such as acetone and alcohols, tend to lead to greater denaturation rates, probably because they remove the solvating water layer from the surface of the enzyme, thereby destabilizing it [40,41]. However, in this work, there was no clear correlation between Im-LipG9 stability and log *P* values, with Im-LipG9 showing significant stability (>80% residual activity) in polar solvents such as ethanol and acetone (Table 3). The lack of correlation becomes clearer when the residual activities of Im-LipG9 are compared for two polar solvents with similar log *P* values, tetrahydrofuran (log *P* 0.48) and ethyl acetate (log *P* 0.68): in these solvents the residual activities were quite different, with values of 82% and 43%, respectively. Similar results have been reported for some other lipases when treated with organic solvents [43–46], so it appears that the specific functional groups and molecular constitution of the solvents are important [45,47].

**Table 3:** Residual activity of immobilized LipG9 (Im-LipG9) after incubation in different organic solvents.

Organic solvents ^a^	log *P*	Residual Activity ^c^ (% ± SEM ^d^)
Control ^b^	***	100
*n*-Heptane	4.0	89 ± 2
*n*-Hexane	3.50	79 ± 2
Cyclohexane	3.2	76 ± 2
Toluene	2.50	86 ± 1
*tert*-Butanol	1.45	71 ± 1
*n*-Butanol	0.80	76 ± 1
Ethyl acetate	0.68	43 ± 2
Tetrahydrofuran	0.46	82 ± 3
*n*-Propanol	0.25	63 ± 3
Acetone	- 0.23	85 ± 1
Ethanol	-0.31	83 ± 3
Acetonitrile	-0.33	72 ± 3
Methanol	-0.76	30 ± 1
Dimethyl sulfoxide	-1.30	0

^a^ Im-LipG9 was incubated in 1 mL of solvent for 8 h at 30°C.

^b^Control without pre-incubation.

^c^ Residual activity was measured by the hydrolysis of triolein in organic media. Conditions: 20 mg of Im-LipG9, 70 mM triolein in *n*-heptane, 30°C, 200 rpm.

^d^ SEM: Standard error of the mean. Assays were carried out in triplicate.

### Regioselectivity of LipG9

**Fig 6:**
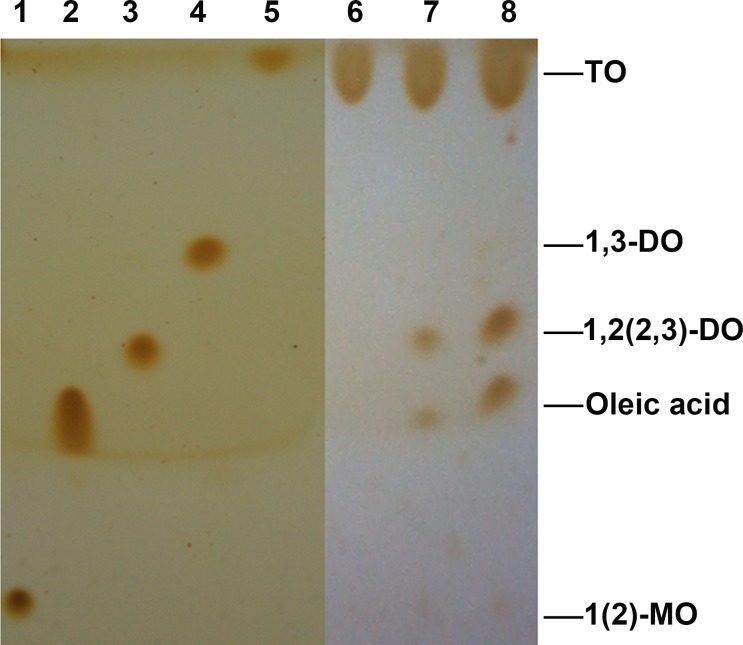
Thin layer chromatography profile of the reagents and products of the hydrolysis of triolein catalyzed by LipG9 in organic medium. Lane 1, 1(2)-monoolein (1(2)-MO); lane 2, oleic acid; lane 3, 1,2(2,3)-diolein (1,2(2,3)-DO); lane 4, 1,3-diolein (1,3-DO); lane 5, triolein (TO); lane 6, the beginning of the reaction; lane 7, products of triolein hydrolysis catalyzed by Fr-LipG9; lane 8, products of triolein hydrolysis catalyzed by Im-LipG9.

Lipases can be classified into two groups based on their positional specificity for the hydrolysis of the ester bond in triglycerides: *sn*-1,3-specific and non-specific [29]. In order to determine the positional specificity of Im-LipG9, hydrolysis assays were performed in *n*-heptane using pure triolein (99%) as the substrate, with 2% (v/v) of water. After 30 min of reaction, the main products present in the medium were oleic acid and 1,2 (2,3)-diolein (Fig. 6). The same profile was observed for the products of the reaction catalyzed by Fr-LipG9 (lane 7, Fig. 6), indicating that the enzyme did not change its positional specificity upon immobilization. Since 1,3-diolein was not detected, LipG9 does not hydrolyze ester bonds at the *sn*-2 position of triacylglycerols and therefore can be classified as a *sn*-1,3-specific lipase [29,48].

### Enantioselectivity of LipG9

The enantioselectivity of Im-LipG9 was evaluated during transesterification of (*R*, *S*)-1-phenyl-1-ethanol and the hydrolysis of its corresponding ester (*R*, *S*)-1-phenylethyl acetate, both in *n*-hexane. For the resolution of (*R*,*S*)-1-phenyl-1-ethanol, Im-LipG9 achieved 49% conversion in 30 h, with high enantiomeric excess of (*R*)-1-phenylethyl acetate (*ee*_*p*_ > 95%), and an enantiomeric ratio (*E*) greater than 200 (Table 4). For the resolution of (*R*, *S*)-1-phenylethyl acetate, Im-LipG9 gave 16% conversion in 60 h, with high enantiomeric excess for (*R*)-1-phenyl-1-ethanol (*ee*_*p*_ > 95%) and an enantiomeric ratio (*E*) greater than 200 (Table 4). The chromatographic profiles observed in reactions catalyzed by Im-LipG9 were the same as those observed in the reactions catalyzed by the lipase CALB (Table 4), confirming the enantiopreference of Im-LipG9 for the *R*-isomer.

**Table 4:** Kinetic resolution parameters from reactions catalyzed by immobilized LipG9 (Im-LipG9) and *Candida antarctica* Lipase B (CALB).

Enzyme	Compound	Time (h)	*c* (%)	*ee*_*s*_(%)	*ee*_*p*_(%)	E
Im-LipG9	(*R*,*S*)-1-phenyl-1-ethanol	30	49	> 95	> 95	> 200
	(*R*, *S*) 1-phenylethyl acetate	60	16	18	> 95	> 200
CALB	(*R*,*S*)-1-phenyl-1-ethanol	2	50	99	99	> 200
	(*R*, *S*) 1-phenylethyl acetate	2	50	99	99	> 200

Reaction conditions: Transesterification reaction: (*R*,*S*)-**1** (0.10 mmol), Im-LipG9 (100 mg) or CALB (20 mg), vinyl acetate (0.2 mL), *n*-hexane (2 mL) and 35°C. Hydrolysis reaction: (*R*,*S*)-**1a** (0.10 mmol), Im-LipG9 (100 mg) or 20 mg (CALB), water (0.2 mL), *n*-hexane (2 mL) and 35°C.

Conversions (*c*) and enantioselectivity (*E*) were calculated from the excesses of substrate (% *ee*_*s*_) and product (% *ee*_*p*_) according to Chen et al. [33].

### Application of LipG9 in ester synthesis

**Fig 7:**
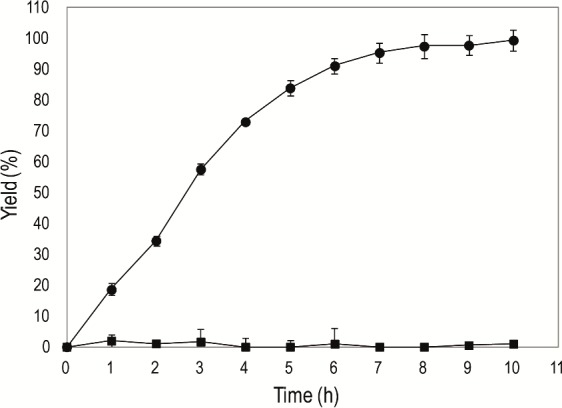
Kinetics of the synthesis of ethyl-oleate in *n*-heptane, catalyzed by different preparations of LipG9. Key: (●) Im-LipG9; (■) lyophilized Fr-LipG9. In all assays, the total activity in the medium was 22 U (as determined by the hydrolysis of triolein in organic media). Conditions: 5 mL *n*-heptane, 70 mM oleic acid, 210 mM ethanol. The mixture was incubated at 37°C and 200 rpm. The error bars represent the standard error of the mean.

The ability of Fr-LipG9 and Im-LipG9 to catalyze the synthesis of ethyl-oleate in *n*-heptane was evaluated using 22 U of total hydrolytic activity in the reaction medium for both preparations. For Im-LipG9, the esterification activity was 16 U per gram of support (U g^-1^) and a yield of 95% was obtained in 7 h, whereas lyophilized Fr-LipG9 was inactive (Fig. 7). These results are in agreement with several studies reporting higher yields in the production of esters using immobilized lipases in relation to free enzymes [49–52].

**Fig 8:**
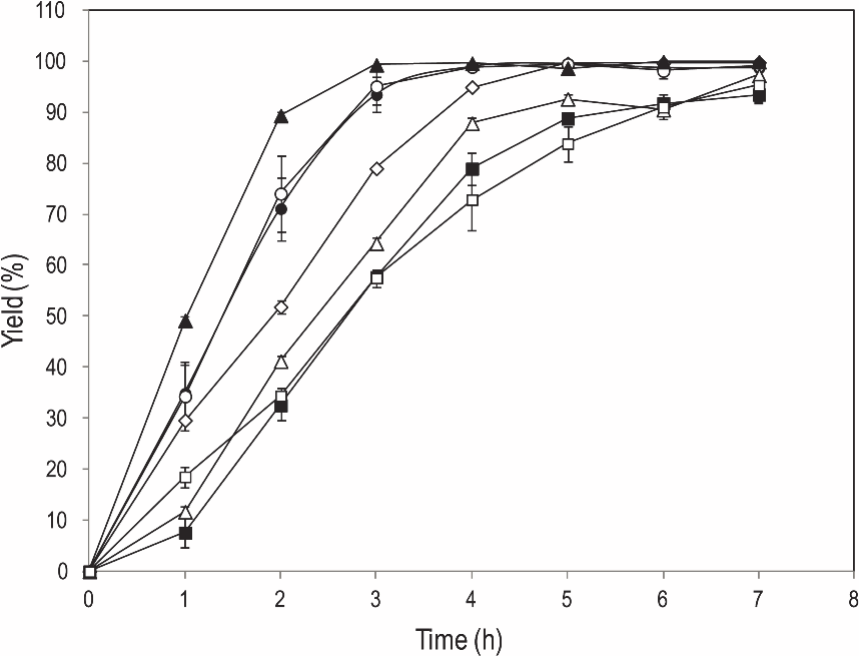
Effect of fatty acid chain length in the yields of esterification reactions catalyzed by immobilized LipG9. Key: (●) caprylic acid (C8); (◊) lauric acid (C12); (○) myristic acid (C14); (▲) palmitic acid (C16); (Δ) stearic acid (C18); (□) oleic acid (C18:1); and (■) linoleic acid (C18:2). In all assays, the total activity in the medium was 22 U (as determined by the hydrolysis of triolein in organic media). Conditions: 5 mL *n*-heptane, 70 mM of fatty acids, 210 mM ethanol. The mixture was incubated at 37°C and 200 rpm. The error bars represent the standard error of the mean.

Further experiments were performed in order to determine the effect of chain length on the esterification activity of Im-LipG9. The best conversions (> 95% in 3 h) were obtained for saturated fatty acids of medium and long chain lengths (C8, C14 and C16), with the maximum activity (29 U g^-1^) being obtained for palmitic acid (C16) (Fig. 8). For long-chain and unsaturated fatty acids (C18, C18:1 and C18:2), conversions of 90% were obtained after 7 h of reaction. Indeed, the esterification activity of Im-LipG9 was not directly related to the length of the acyl chain.

## Discussion

In the current work, we immobilized LipG9, complexed with its specific foldase (LifG9), on the hydrophobic support Accurel by physical adsorption and studied its potential in several model reactions in water-restricted media. Not only is this the first study of the immobilization of a lipase-foldase complex obtained from metagenomics, but also there are only few studies involving lipases complexed with their specific foldases and they are normally devoted to investigation of strategies for co-expression *in vitro* and *in vivo* in order to achieve expression of active enzymes [53–55].

The only previous study of immobilization of a lipase-foldase complex was that of Peng et al. [56], for the lipase-foldase complex from *Pseudomonas aeruginosa* CS2 immobilized on Celite-54. Although the systems are quite different, our immobilized preparation gave better esterification results than did theirs: Our best conversion, of close to 100% in 3 h for the synthesis of ethyl palmitate in *n*-heptane (Fig. 8) was obtained much faster than their best result, a conversion of 98% after 10 h for the synthesis of butyl-acetate in *n*-heptane.

In the particular case of the use of lipases from metagenomics to catalyze reactions in organic media, there are only relatively few studies and Im-LipG9 compares quite favorably. The first metagenome-derived lipase that was shown to have a significant esterification activity in solvent-free medium (0.12 U mg^-1^ for the synthesis of 1-propyl laurate) was LipS, isolated by Chow et al. [20]. More recently, Brault et al. [57] demonstrated the potential of using whole-cell biocatalyst with a recombinant metagenomic lipase (LipIAF5–2) for the synthesis of short-chain flavor esters. In our case, Im-LipG9 catalyzes the synthesis of ethyl esters of fatty acids of various chain lengths and degrees of unsaturation (C8, C12, C14,C16, C18, C18:1, C18:2) in *n*-heptane, in all cases giving yields above 85% after 7 h of reaction.

In the current work, Im-LipG9 showed good stability, with over 80% residual activity after 8 h incubation in various pure organic solvents, among them acetone and ethanol. No other lipase obtained through metagenomics exhibits satisfactory stability when incubated for 8 h in 100% acetone or ethanol: Two previously isolated metagenomic lipases, LipIAF1–6 [58] and LipC12 [18], only showed good stability in acetone and ethanol at solvent concentrations up to 30% (v/v). In fact, the good solvent stability of Im-LipG9 may, in part be due to the fact that it was co-immobilized with its foldase, which may help it to maintain its tertiary structure in conditions that might otherwise denature it.

The high triolein-hydrolyzing activity in organic medium of Im-LipG9 in comparison to the free enzyme (Table 2) corresponds to retention of activity values (R) well above 150% for all protein loadings. The suggested explanation for this phenomenon is that the lipase is adsorbed in its open structural conformation, in other words, the lipase simultaneously binds to the support and undergoes interfacial activation [49]. In our work, this activation was facilitated by the hydrophobicity of Accurel and by the fact that LipG9 has a hydrophobic lid sub-domain [21].

Im-LipG9 appears to have good potential for application in media containing organic solvents. Its enantioselectivity is comparable to those of commercial lipases from *Candida antarctica* B (CALB), *Pseudomonas cepacia* and *P*. *fluorescens*, which show enantiomeric ratios greater than 200 in the resolution of 1-phenyl-1-ethanol [59]. Of particular interest is the stability presented by Im-LipG9 in hydrophilic solvents such as ethanol and acetone, since such solvents are used in many organic synthesis reactions and most lipases, including commercial formulations such as Novozym 435, have poor stability in these solvents [60].

In conclusion, we have reported the most thorough characterization to date of the potential for the application of a metagenomic lipase in organic solvents. The combined characteristics of Im-LipG9, namely its good activity and stability in organic solvents, along with its regioselectivity and enantioselectivity, make Im-LipG9 an interesting prospect for use in biocatalysis.

### Nucleotide sequence accession number

The *lipG9* and *lifG9* nucleotide sequences reported here are available in the GenBank database under the accession numbers GenBank: KM023399 and GenBank: KM023400, respectively.

## Supporting Information

S1 Text Synthesis and characterization of racemates used to determine the enantioselectivity of LipG9. (DOCX)
