# Supplementary material for: Immobilization and Characterization of a New Regioselective and Enantioselective Lipase Obtained from a Metagenomic Library
Source: PLoS One. 2015 Feb 23;10(2):e0114945. doi: 10.1371/journal.pone.0114945 (PMC4338019; doi:10.1371/journal.pone.0114945)
Supplement: S1 Text — (DOCX) [file pone.0114945.s001.docx]

***Supporting Information***

**Text S1. Synthesis and characterization of racemates used to determine the enantioselectivity of LipG9**

*Synthesis of the racemate (R,S)-1-phenyl-1-ethanol*

NaBH_4_ (2.0 g, 55 mmol) was added to a solution of acetophenone (6.0 g, 50 mmol) in methanol (50 mL) and the reaction was maintained under magnetic agitation at room temperature for two hours. The methanol was evaporated under reduced pressure and water was added to the residue, with the pH of the solution then being adjusted to 6.0 with aqueous HCl 1 mol L^-1^. In sequence, the aqueous phase was extracted with dichloromethane (3 × 10 mL), the combined extracts were dried with anhydrous magnesium sulfate and filtered off. After evaporation of the dichloromethane, the product was purified by column chromatography on silica gel (eluent: *n*-hexane:ethyl-acetate 9:1) and characterized. Yield: 4.9 g (80%); ^1^H NMR (200 MHz, CDCl_3_, TMS), δ (ppm): 1.42 (d; *J* = 6.5 Hz; 3H); 2.70 (s; 3H); 4.79 (q; *J* = 6.5; 1H); 7.29 (m; 5H). ^13^C NMR (50 MHz, CDCl_3_), δ (ppm): 25.1; 70.2; 125.4; 127.3; 128.4; 145.8. IR (cm^-1^): 3349, 2965, 1882, 1489, 1448, 1384, 1077, 760, 699. MS (70 eV), m/z (relative intensity): 122 (M+, 34); 107 (90); 79 (100); 77(54); 51 (21); 43 (24).

*Synthesis of racemate (R, S) 1-phenylethyl acetate*

Acetic anhydride (5.1 g, 50 mmol) was added to a solution of 1-phenyl-1-ethanol (3.1 g, 25 mmol) in pyridine (15 mL) and the reaction was maintained under magnetic stirring at room temperature overnight. The mixture was then diluted with ethyl-acetate (20 mL) and washed with aqueous saturated copper sulfate to remove the pyridine. The organic phase was dried over anhydrous magnesium sulfate, filtered off and the solvent evaporated under reduced pressure. The product was purified by column chromatography on silica gel (eluent: *n*-hexane:ethyl-acetate 9:1) and characterized.

Yield: 3.0 g (75%). ^1^H NMR (200 MHz, CDCl^3^, TMS), δ (ppm): 1.52 (d; *J* = 6.6 Hz; 3H); 2.06 (s; 3H); 5.88 (q; *J* = 6.6; 1H); 7.33 (m; 5H). ^13^C RMN (50 MHz - CDCl_3_), δ (ppm): 21.3; 22.1; 72.2; 126.0; 127.8; 128.4; 141.6; 170.2. IR (cm^-1^): 3029, 2969, 1749, 1500, 1362, 1253, 1075.EM (70 eV), m/z (relative intensity): 164 (M+, 23); 122 (100); 105 (67); 104 (89); 107 (36); 51 (15); 43 (55).

^1^H and ^13^C NMR spectra were recorded on a Bruker DPX200 spectrometer. The chemical shifts of ^1^H and ^13^C NMR signals are quoted relative to internal CHCl_3_ (ä_H_ = 7.26) and CDCl_3_ (ä_C_ =77.00) or tetramethylsilane (ä_H_ = 0.0). ^1^H NMR data are reported as follows: chemical shift in ppm (ä), multiplicity (s = singlet, d = doublet, t = triplet, q = quartet, m = multiplet, brs = broad singlet, etc.), coupling constant (Hz) and relative intensity. ^13^C NMR data are reported as follows: chemical shift in ppm (ä). The GC-MS analyses were performed on a Shimadzu GC-MS QP-5050A under EI conditions, with a capillary column SLBS 5 ms, with electron impact ionization (70 eV), mode split injector at 250°C, with temperature program starting at 50°C for 1 min, ramp 7°C min^-1^ to 250°C for 10 min with a flow of helium 1 mL min^~~-~~1^. IR spectra were recorded on a Bomem MB100 spectrometer.
